# Supplementary material for: The Combined Cultivation of Feruloyl Esterase-Producing Strains with CMCase and Xylanase-Producing Strains Increases the Release of Ferulic Acid
Source: Microorganisms. 2022 Sep 22;10(10):1889. doi: 10.3390/microorganisms10101889 (PMC9610611; doi:10.3390/microorganisms10101889)
Supplement: Supplementary file 1 [file microorganisms-10-01889-s001.zip › microorganisms-1914708-supplementary.pdf]

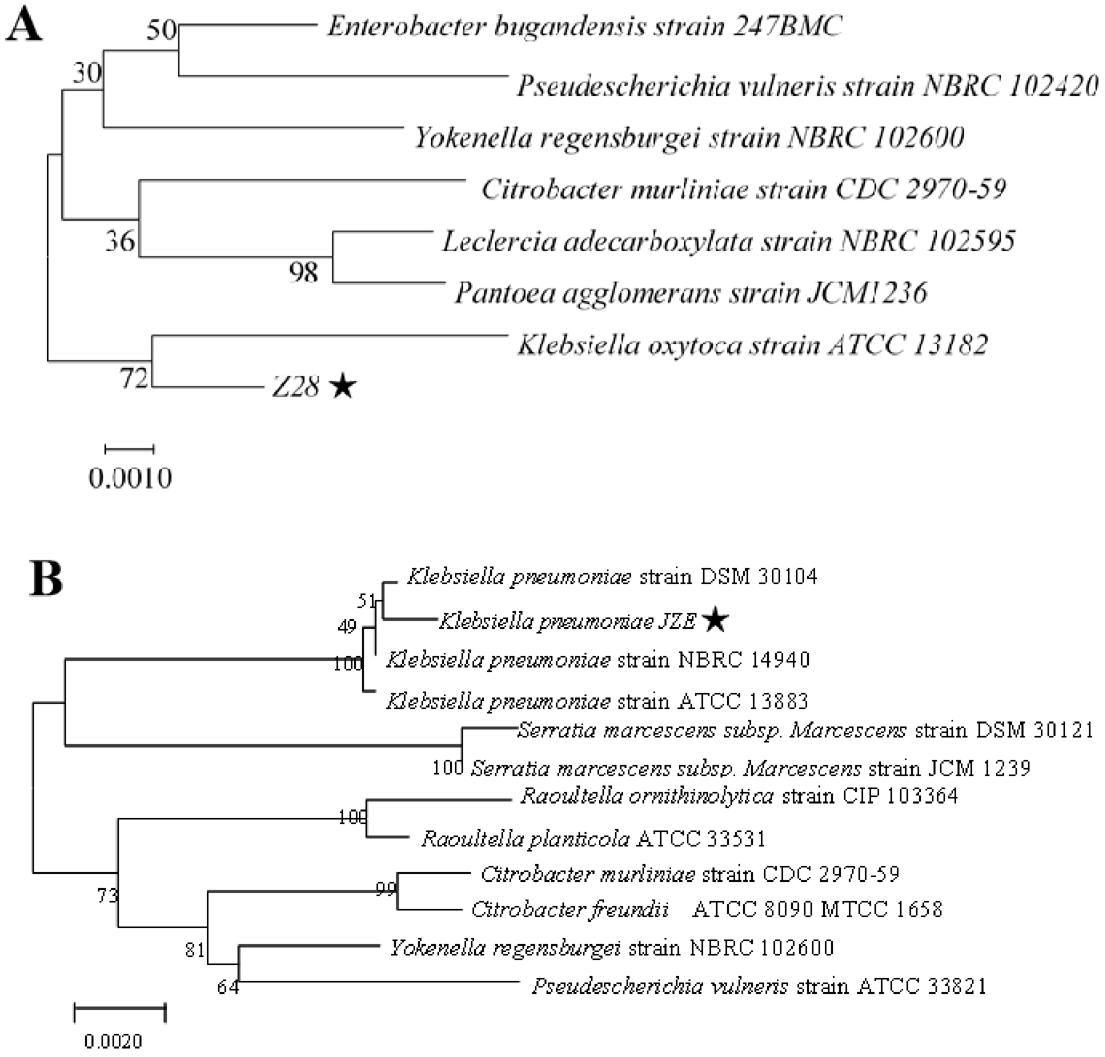
A
Enterobacter bugandensis strain 247BMC
Pseudescherichia vulneris strain NBRC 102420
Yokenella regensburgei strain NBRC 102600
Citrobacter murliniae strain CDC 2970-59
Leclercia adecarboxylata strain NBRC 102595
Pantoea agglomerans strain JCM1236
Klebsiella oxytoca strain ATCC 13182
Z28 ★
50
30
36
98
72
0.0010
B
Klebsiella pneumoniae strain DSM 30104
Klebsiella pneumoniae JZE ★
Klebsiella pneumoniae strain NBRC 14940
Klebsiella pneumoniae strain ATCC 13883
Serratia marcescens subsp. Marcescens strain DSM 30121
Serratia marcescens subsp. Marcescens strain JCM 1239
Raoultella ornithinolytica strain CIP 103364
Raoultella planticola ATCC 33531
Citrobacter murliniae strain CDC 2970-59
Citrobacter freundii ATCC 8090 MTCC 1658
Yokenella regensburgei strain NBRC 102600
Pseudescherichia vulneris strain ATCC 33821
51
49
100
100
100
73
99
81
64
0.0020

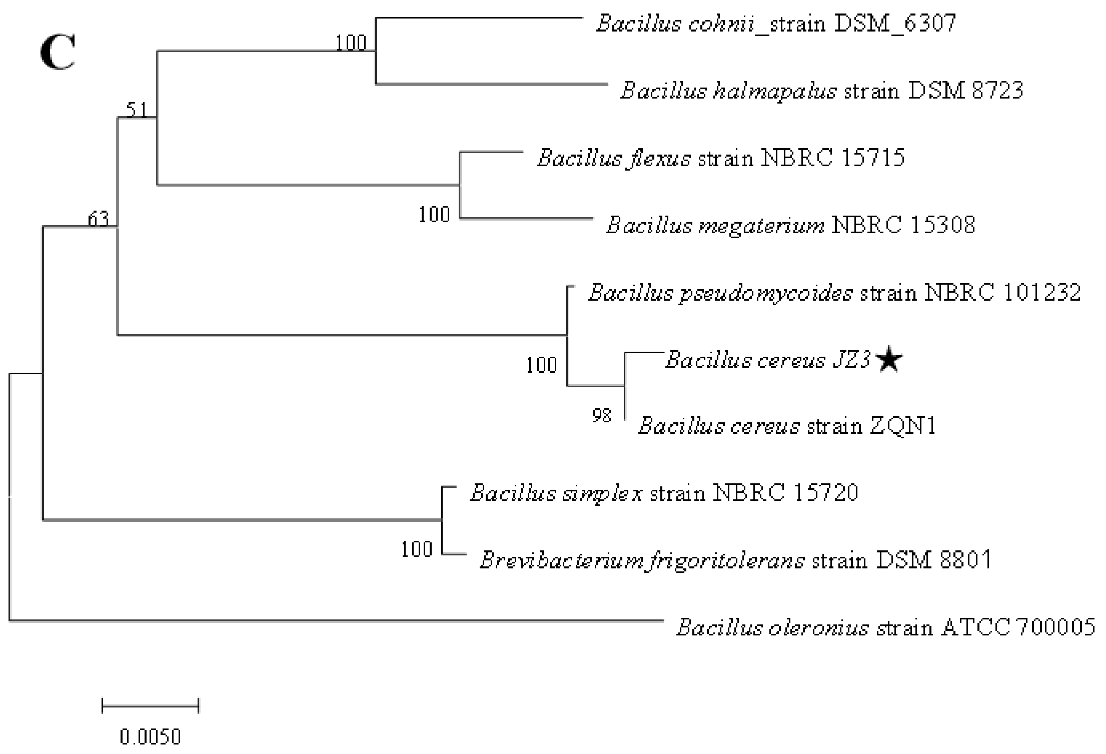
C
Bacillus cohnii_strain DSM_6307
Bacillus halmapalus strain DSM 8723
Bacillus flexus strain NBRC 15715
Bacillus megaterium NBRC 15308
Bacillus pseudomycoides strain NBRC 101232
Bacillus cereus JZ3 ★
Bacillus cereus strain ZQN1
Bacillus simplex strain NBRC 15720
Brevibacterium frigoritolerans strain DSM 8801
Bacillus oleronius strain ATCC 700005
100
51
100
63
100
98
100
0.0050

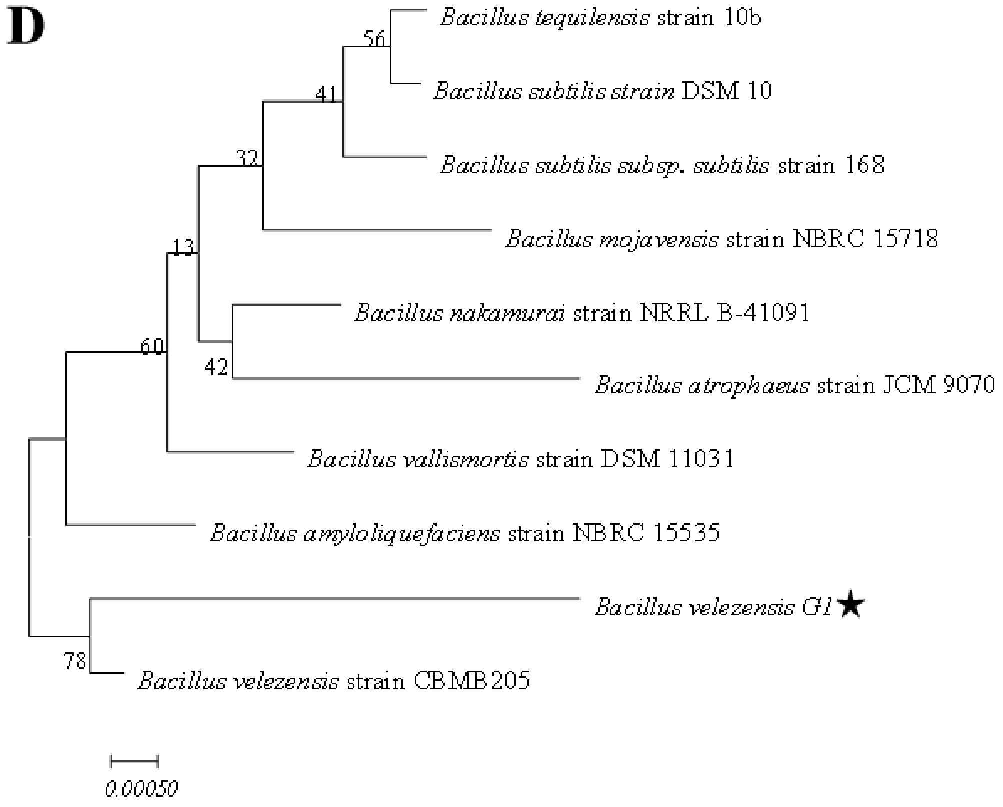
D
Bacillus tequilensis strain 10b
Bacillus subtilis strain DSM 10
Bacillus subtilis subsp. subtilis strain 168
Bacillus mojavensis strain NBRC 15718
Bacillus nakamurai strain NRRL B-41091
Bacillus atrophaeus strain JCM 9070
Bacillus vallismortis strain DSM 11031
Bacillus amyloliquefaciens strain NBRC 15535
Bacillus velezensis G1 ★
Bacillus velezensis strain CBMB205
56
41
32
13
60
42
78
0.00050

E

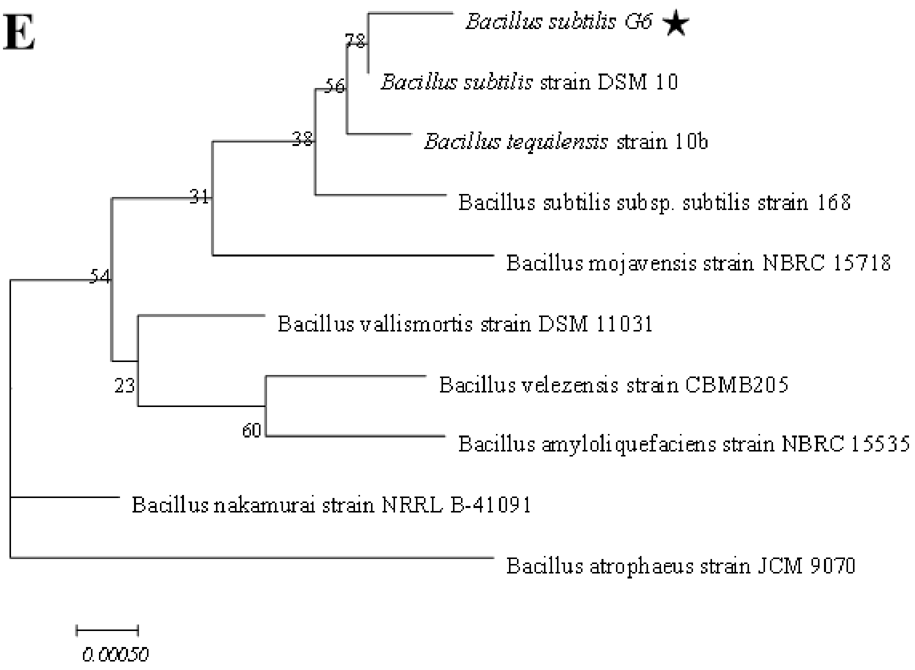

Figure S1. Identification of Feruloyl Esterase-producing strains(A) with and Xylanase-producing Strains(B-C), CMCase-producing Strain*s*(D-E) by 16SrRNA sequencing after constructing the phylogenetic tree using the Neighbor-joining method at 500 replicates of bootstrap value

Table S1.Published microorganism using alignments in the work

strain	References
Klebsiella pneumoniae JZE	1
Siccibacter colletis G2	2

References

1. Yao Zhang , J., Xian Zhang ,Zhiping Feng, Lijuan Yang, Screening ofAlkaline Tolerant Xylanase Strains in Bamboo Rat Intestines and Study on Enzymatic Properties. *Science and Technology of Food Industry* **2021,**

42 (23), 118-125.

2. Wei Guo, Y. Z., Xian Zhang,Lijuan Yang, Screening of acid cellulase strain degradation resistance in bamboo rats and study on enzymatic properties. *Feed Research* **2021,** *44* (17), 65-69.
